# Dysregulated Maresin Concentrations in Plasma and Nasal Secretions From Patients With Chronic Rhinosinusitis

**DOI:** 10.3389/fimmu.2021.733019

**Published:** 2021-08-31

**Authors:** Issa Beegun, Duco S. Koenis, Ghassan Alusi, Jesmond Dalli

**Affiliations:** ^1^William Harvey Research Institute, Barts and The London School of Medicine and Dentistry, Queen Mary University of London, London, United Kingdom; ^2^Centre for Inflammation and Therapeutic Innovation, Queen Mary University of London, London, United Kingdom

**Keywords:** resolving, resolution, inflammation, phagocytes, omega-3, chronic rhinosinusitis, airway inflammation

## Abstract

The mechanisms that lead to disease onset and propagation in patients with chronic rhinosinusitis (CRS) are not fully elucidated. Maresins (MaR) are a family of essential fatty acid-derived lipid mediators that play a central role in the regulation of inflammation with several studies demonstrating that these mediators display protective activities in airway inflammation. Therefore, in the present studies we evaluated whether concentrations of these mediators were altered in both peripheral blood and nasal secretions from CRS patients. Herein, we focused on patients with CRS that also develop nasal polyps (CRSwNP), given that therapeutic options for the treatment of these patients are limited. Thereby, insights into disease mechanisms in these patients may help design more effective treatments. For this purpose, we compared maresin concentrations from CRSwNP patients with those found in healthy volunteers or patients with an upper respiratory tract infection (URTI), as a self-resolving inflammatory condition. Using liquid chromatography tandem mass spectrometry, we found that MaR concentrations were significantly decreased in plasma from patients with CRSwNP when compared to healthy volunteers. MaR concentrations were observed to be significantly upregulated in nasal secretions from patients with CRSwNP when compared with both healthy volunteers and URTI subjects. Concentration of these mediators in both plasma and nasal secretions from CRSwNP patients were positively correlated with quality-of-life scores in these patients. Assessment of the concentrations of other pro-resolving and pro-inflammatory lipid mediators (LM) demonstrated that there was a general shift in LM levels in both plasma and nasal secretions from CRSwNP when compared with healthy volunteers and URTI subjects. Of note, incubation of peripheral blood cells from CRSwNP patients with MaR1 downregulated the expression of activation markers on peripheral blood phagocytes, including CD41 and CD62P, markers of platelet-leukocyte heterotypic aggregates. Together these findings demonstrate that both local and systemic LM concentrations, in particularly those of the MaR family, become altered in patients with CRSwNP. They also suggest that therapeutics designed around MaR1 may be useful in regulating the activation of phagocytes in patients with CRSwNP thereby potentially also limiting the local inflammatory response in these patients.

## Introduction

Chronic rhinosinusitis (CRS) is a common inflammatory disorder of the respiratory tract resulting in symptoms of nasal blockage, discharge, loss of smell and facial pain lasting for over 12 weeks (1). It is estimated that 2.1% of the adult population in the USA suffer with CRS (2). In Europe studies estimate the prevalence of CRS to be between 2.1% and 4.3% (3–5). Approximately 20% of patients with CRS develop nasal polyps (CRSwNP) (6). This condition affects 11% of the UK adult population with 120,000 secondary care outpatient encounters and 40,000 sinus operations per year in England and Wales alone (7). Morbidity is considerable, affecting physical, social and emotional health. In addition to nasal symptoms, patients suffering from CRS report lower quality of life scores with adverse effects on sleep quality, sexual function, work productivity and mental health. The quality of life for patients suffering with CRS is thought to be worse than patients suffering with back pain or heart failure (8–10).

CRS with nasal polyps (CRSwNP) is a particularly difficult to treat aggressive phenotype associated with asthma and aspirin intolerance (11). It remains uncertain whether this population of patients represent the severe end of the CRSwNP disease spectrum or whether a unique mechanism underlies the pathogenesis of this aggressive form of disease (1). Importantly, to date establishing whether a patient with CRSwNP is intolerant to NSAIDs remains a challenging task (1, 12, 13). Several approaches including systemic and local NSAID challenges are available (14, 15). However, medical supervision with access to resuscitation equipment and overnight hospital admission are recommended, highlighting the need for further research to identify biomarkers and the development of better diagnostic tests for the identification and stratification of patients with CRSwNP. One key aspect that has hindered the development of such tests is our limited understanding of the cellular and molecular mechanism operative in patients with CRSwNP.

Chronic inflammation arises from the inability of the host’s immune response to limit the production of inflammatory factors. It is now well established that the specialised pro-resolving mediators (SPM) are central in orchestrating the cessation of inflammation (16–18). These autacoids are formed *via* the conversion of essential fatty acids, including docosahexaenoic acid and n-3 docosapentaenoic acid by specific biosynthetic enzymes to yield structurally distinct mediators. The SPM are divided into four main families the resolvins, protectins maresins and lipoxins (16–18). These autacoids families share defining biological actions that contribute to the resolution of inflammation. These include: 1) counter-regulating the production of inflammatory mediators, 2) limiting of leukocyte trafficking to the site of inflammation and 3) upregulating the clearance of apoptotic cells and cellular debris by macrophages (16–18). Furthermore, each of these autacoids displays characteristic biological actives, for example resolvin (Rv) D2 regulates nitric oxide formation in endothelial cells (19), whereas the DHA-derived protectins limit viral replication (20). Findings made in both experimental systems and humans demonstrate that disruptions in the formation and activity of these autacoids contribute to the exacerbation of inflammation (16–18).

Recent studies assessing the levels of select SPM in sinus tissues from patients with CRSwNP suggest that the production of RvD2 and Lipoxin A_4_ may become altered in these patients when compared to either CRS patients without polyps or healthy volunteers and correlates to disease severity (21). Chronic inflammatory conditions are characterized by systemic inflammation and recent findings suggest that plasma SPM concentrations may reflect disease activity in peripheral tissues (22). Therefore, in the present study, we sought to establish whether systemic SPM concentrations are also altered in patients with CRSwNP. Furthermore, several reports suggest that an infective stimulus in the form of an upper respiratory tract infection (URTI) may act as a trigger (23). Thus, to gain insights into the mechanisms that lead to the transition from a healthy state to CRSwNP we compared SPM levels with those from both healthy volunteers and patients with an active URTI.

## Methods

After informed consent was obtained each participant completed a copy of the Sino-Nasal Outcome Test-22 (SNOT-22), Nasal Obstruction Symptom Evaluation (NOSE) and 36-Item Short Form Survey (SF-36). The SNOT-22 is a questionnaire consisting of 22 questions evaluating a range of symptoms experienced by suffers of nasal disease (24). The NOSE questionnaire consists of five questions which predominantly focus on evaluating breathing difficulties (25). The SF-36 has been reported as the most widely used health-related quality of life survey instrument in the world (26). It is comprised of 36 items that assess eight health concepts has been used to determine the cost-effectiveness of a health treatments.

### Ethics

Participants with a history of CRSwNP with failed medical management, healthy controls with no previous ear, nose and throat disease as well as those undergoing nasal cavity surgery due to non-inflammatory conditions (cosmetic or post traumatic deformity; septoplasty or septorhinoplasty) and participants with URTI were recruited. (Research Ethics Committee Number: 17/EM/0447).

### Blood Collection for Lipid Mediator Profiling

Whole blood was collected from a peripheral vein of the upper limb using a 21g butterfly needle connected to a Becton Dickinson Vacutainer^®^ containing 3.2% trisodium citrate. Samples were then centrifuged at 2500 x g for 10 minutes and 1.5 ml of the plasma supernatant transferred to 15 ml Falcon^®^ tubes containing 6 ml of ice-cold methanol with internal standards (500 pg of each; 5S-HETE-d_8_, LTB_4_-d_4_, LXA_4_-d_5_, RvD2-d_5_ and PGE_2_-d_4_).

### Nasal Secretion Lipid Mediator Profiling

Nasal secretions were collected using a polyurethane sponge (RG 27 grau, Gummi-Welz GmbH & Co., Germany), pre-cut into 20 × 15 × 5 mm pieces and sterilized by autoclaving for 20 min at 121°C prior to use. One sponge piece was placed into the anterior nasal cavity under direct vision using a head mounted light and Tilley’s nasal dressing forceps. Sponges were left in place for 10 minutes, removed and placed into 1 ml of ice-cold methanol with internal standards. Samples were stored at -80°C until further analysis.

Prior to lipid mediator extraction, stored sponges were placed into a modified 1.5 ml Eppendorf™ tube. Modification consisted of removing 5 mm from the lower portion of the tube. This was accomplished by cutting the tube at an angle using a pair of Mayo scissors. The modified Eppendorf containing the sponge was suspended in a 15 ml Falcon^®^ tube using Prolene^®^ 4-0 suture (Ethicon Polypropylene Suture). The cap of the Falcon^®^ tube was used to secure the sutured modified Eppendorf during centrifugation at 2000 x g for 10 minutes. Post centrifugation the supernatant was transferred to 15 ml Falcon^®^ tubes and stored at -80°C for subsequent lipid mediator profiling. The pellet was allowed to stand under a sterile hood for ten minutes to allow evaporation of any remaining methanol. The pellet was then suspended in 400 μl of Milli-Q^®^ water and stored at -80°C until protein quantification was performed.

### Lipid Mediator Profiling

Samples were extracted and lipid mediators were identified and quantified as described (22). In brief, plasma was collected as described above and immediately placed on dry ice in 4 volumes of ice-cold methanol containing deuterated internal standards (d_4_-LTB_4_, d_5_-LXA_4_, d_4_-PGE_2_, d_5_-RvD2) representing each chromatographic region of identified LM. Following protein precipitation (-20°C for a minimum of 45 min), supernatants were extracted on an ExtraHera instrument (Biotage) using solid-phase extraction with Isolute C18 500mg columns (Biotage). Methyl formate and methanol fractions were collected, brought to dryness and resuspended in phase (methanol/water, 1:1, vol/vol) for injection on a Shimadzu LC-20AD HPLC and a Shimadzu SIL-20AC autoinjector, paired with a QTrap 5500 or QTrap 6500+ (Sciex). In the analysis of mediators eluted in the methyl formate fraction, the QTRAP 5500 was operated in negative ion mode using a multiple reaction monitoring method. An Agilent Poroshell 120 EC-C18 column (100 mm × 4.6 mm × 2.7 μm) was kept at 50°C and mediators eluted using a mobile phase consisting of methanol/water/acetic acid of 20:80:0.01 (vol/vol/vol) that was ramped to 50:50:0.01 (vol/vol/vol) over 0.5 min and then to 80:20:0.01 (vol/vol/vol) from 2 min to 11 min, maintained till 14.5 min and then rapidly ramped to 98:2:0.01 (vol/vol/vol) for the next 0.1 min. This was subsequently maintained at 98:2:0.01 (vol/vol/vol) for 5.4 min, and the flow rate was maintained at 0.5 ml/min. In the analysis of mediators eluted in the methanol fraction, the QTrap 6500+ was operated in positive ion mode using a multiple reaction monitoring method. An Agilent Poroshell 120 EC-C18 column (100 mm × 4.6 mm × 2.7 μm) was kept at 50°C and mediators eluted using a mobile phase consisting of methanol/water/acetic acid 55:45:0.5 (vol:vol:vol) over 5 min, that was ramped to 80:20:0.5 (vol:vol:vol) for 2 min, maintained at 80:20:0.5 (vol:vol:vol) for the successive 3 min and ramped to 98:2:0.5 (vol:vol:vol) over 3 min. This condition was maintained for 3 min. Each lipid mediator was identified using established criteria, including: (1) presence of a peak with a minimum area of 2000 counts, (2) matching retention time to synthetic or authentic standards, (3) >4 data points, (4) signal to noise ratio ≥ 3. Furthermore, to confirm the identity of these mediators in representative samples we obtained MS/MS spectra, matching of at least 6 diagnostic ions to that of reference standard, with a minimum of one backbone fragment being identified. Calibration curves were obtained for each mediator using synthetic compound mixtures at 0.78, 1.56, 3.12, 6.25, 12.5, 25, 50, 100, and 200 pg that gave linear calibration curves with R^2^ values of 0.98–0.99. These calibration curves were then used to calculate the abundance of each mediator per 1mL of plasma for each individual sample.

### Whole Blood Assays

Participants with a history of failed medical and surgical management of CRSwNP were recruited and peripheral blood was collected between 10 am and 12 pm (Research Ethics Committee Number: 17/EM/0447) from a peripheral vein of the upper limb as described above. Forty-five microliters of whole blood were transferred to a 15 ml Falcon^®^ tube and incubated with MaR1 at either 1 nM or 10 nM or vehicle (PBS+0.01% Ethanol). Whole blood samples were kept at 37 ^0^C for 45 minutes. The blood was then washed using PBS ^-/-^ containing 0.02% BSA and then incubated with the following antibodies: APC-Cy7 conjugated mouse anti human CD16 (Clone 3G8, Biolegend), BV785 conjugated mouse anti human CD14 (Clone M5E2, Biolegend)m BV711 conjugated mouse anti human CD11b (Clone ICRF44, Biolegend), conjugated mouse anti human CD16 (Clone 3G8, Biolegend), AF700 conjugated mouse anti human CD45 (Clone 2D1, Biolegend), PerCP-Cy7 conjugated mouse anti human CD66b (Clone G10F5, Biolegend), PE conjugated mouse anti human Siglec 8 (Clone 7C9, Biolegend), eFluor 450 conjugated mouse anti human CD41 (Clone H1P8, Biolegend), PE-Cy5 conjugated mouse anti human CD62P (Clone AK4, Biolegend) and PE-CD594 conjugated mouse anti human CD49d (Clone 3G8, Biolegend) at room temperature for 15 minutes. Ten microliters of counting beads were then added and samples washed with PBS ^-/-^ containing 0.02% BSA. Red Blood cells were then lysed and fixed using Red-Blood Cells lysis and fixation buffers (Beckman Coulter). Cells were then suspended in PBS ^-/-^ containing 0.02% BSA and staining evaluated using LSR Fortessa cell analyser (BD Biosciences) and analysed using FlowJo software (Tree Star Inc., V10).

In select experiments blood was first incubated with MaR1 as detailed above for 15 minutes at 37°C, then with platelet activating factor (PAF; 100nM) for 30 minutes at 37°C. Cells were stained, fixed and staining was evaluated as detailed above.

### Data Analysis and Statistical Testing

Flow cytometry data was analysed using FlowJo v10.4. For Partial Least Square Discriminant analyses (PLS-DA), missing values were replaced by 1/5^th^ of the minimum value of each variable across the samples, and features with a constant or single value across samples were removed. Data was then auto-scaled, 2D plots of Component 1 and 2 from each PLS-DA with their 95% confidence intervals (CI) were constructed, Variable Importance in Projection (VIP) scores were calculated, and internal validation by leave-one-out cross-validation and calculation of a coefficient of determination (R^2^) for each model was performed using MetaboAnalyst (27). Mahalanobis distances between group centers and Hotelling’s T-squared test p-values were calculated for Component 1 and 2 scores of the PLS-DA models using *pca-utils* (28). All other statistical tests were performed using GraphPad Prism 9 and Microsoft Excel

## Results

### Increased Disease Severity and Decreased QoL in Patients With CRSwNP

A total of 51 participants were recruited into the present study. 16 of these participants were healthy volunteers, 27 had CRSwNP and 8 had an active URTI. None of the 8 participants with an active URTI required medical attention or prescription medication. The 27 participants with CRSwNP all had a history of failed medical treatment requiring surgical intervention (see **Table 1** for patient information).

**Table 1:** Participant demographics.

Group median age	Control n=16		CRSwNP n=27		URTI n=8
	31		51		32
	Plasma n=15	Nasal secretions n=4	Plasma n=24	Nasal secretions =16	Plasma & nasal secretions n=8
Median age	31	29	47	49	32
Gender F:M	F7:M8	F1:M3	F9:M14	F6:M10	F4:M4
Allergies			2	1	
Asthma			4	2	
Aspirin Sensitivity			2	1	
Previous Surgery			23	2	1
Smoking					
Saline Wash			4	2	
Topical Steroids			3	1	
Systemic Steroids			1		
Antibiotics			1		
Regular medication			3	2	1
PRN medication			3	2	

Participant demographics. Of the 16 healthy participants none were on regular medication or had a past medical history involving the ear, nose and throat region of the body. Of participants with CRSwNP all required surgical intervention as a result of persistent disease with poor quality of life following medical intervention. 8 of the CRSwNP participants used topical treatments. One participant had received a course of systemic steroids and antibiotics in the preceding 8 weeks prior to recruitment. Seven participants had a history of asthma and 4 reported aspirin sensitivity.

**Figure 1:**
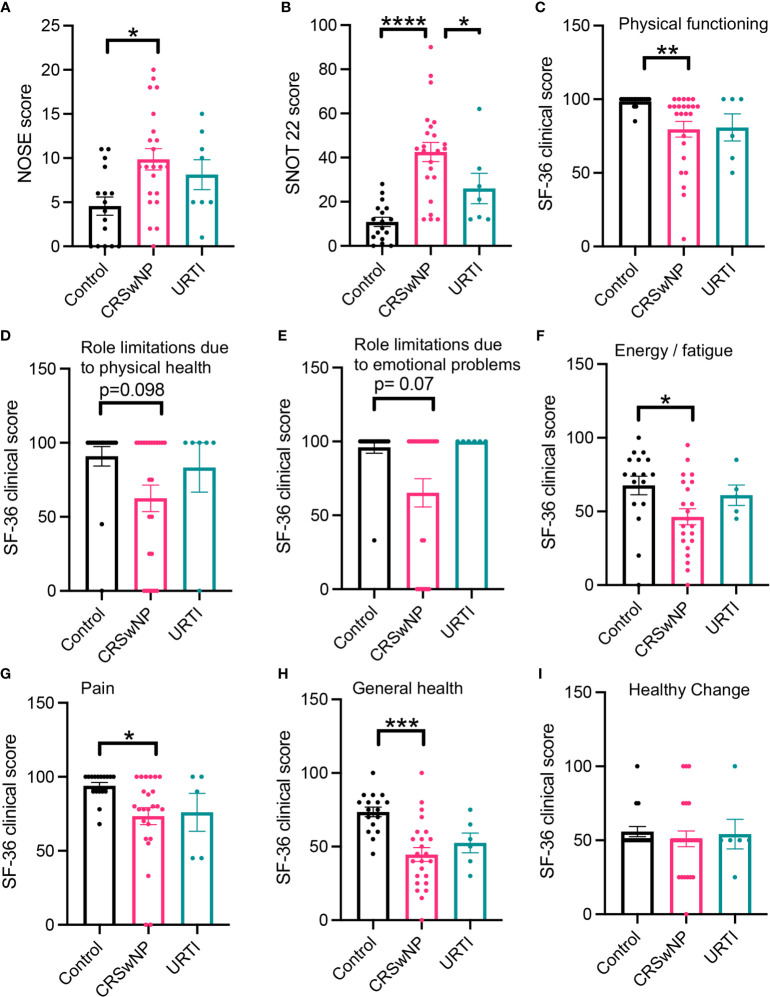
Participants with CRSwNP have increased nasal cavity clinical scores and report poor quality of life. Compared with healthy participants and those who have recovered from an URTI, CRSwNP report worse nasal cavity complaints **(A, B)**. CRSwNP also report comparatively lower QoL scores **(C–I)**. Kruskal-Wallis test used to identify differences between the mean ranks of at least one pair of the groups. Dunn’s pairwise tests was carried out to identify differences between group pairs. *p < 0.05, **p = < 0.01, ***P < 0.001, ****p < 0.0001.

We first evaluated parameters linked with both quality of life (QoL) and disease severity and found that, in line with previous findings (8, 23), patients with CRSwNP reported increased disease severity scores and reduced QoL. This is characterized by increased NOSE scores and SNOT22 scores (**Figures 1A, B**, Dunn’s multiple comparisons test, p= 0.029; Dunn’s multiple comparisons test, p = <0.0001) when compared with healthy volunteers and/or patients with URTI. Physical functioning, physical health, emotional and energy levels were also reduced in participants with CRSwNP when compared to controls (**Figures 1C–F**). Compared with controls CRSwNP participants also reported increased pain scores (**Figure 1G**) and reduced general health scores (**Figures 1H, I**). Of note, while we also observed trends towards a reduction in a number of the parameters evaluated in patients with URTI, these did not reach statistical significance.

### Plasma Maresin Concentrations Correlate With Quality-of-Life Measures and Disease Severity Scores

Maresins display potent immune regulatory activities with members of this family also exerting protective actions in airway inflammation (29, 30). Therefore, we next evaluated whether plasma concentrations of these protective autacoids were altered in plasma and nasal secretions from CRSwNP patients when compared to both healthy volunteers and patients with URTI. We also sought to evaluate whether the concentrations of these molecules were linked with disease severity scores and QoL measures in these patients.

**Figure 2:**
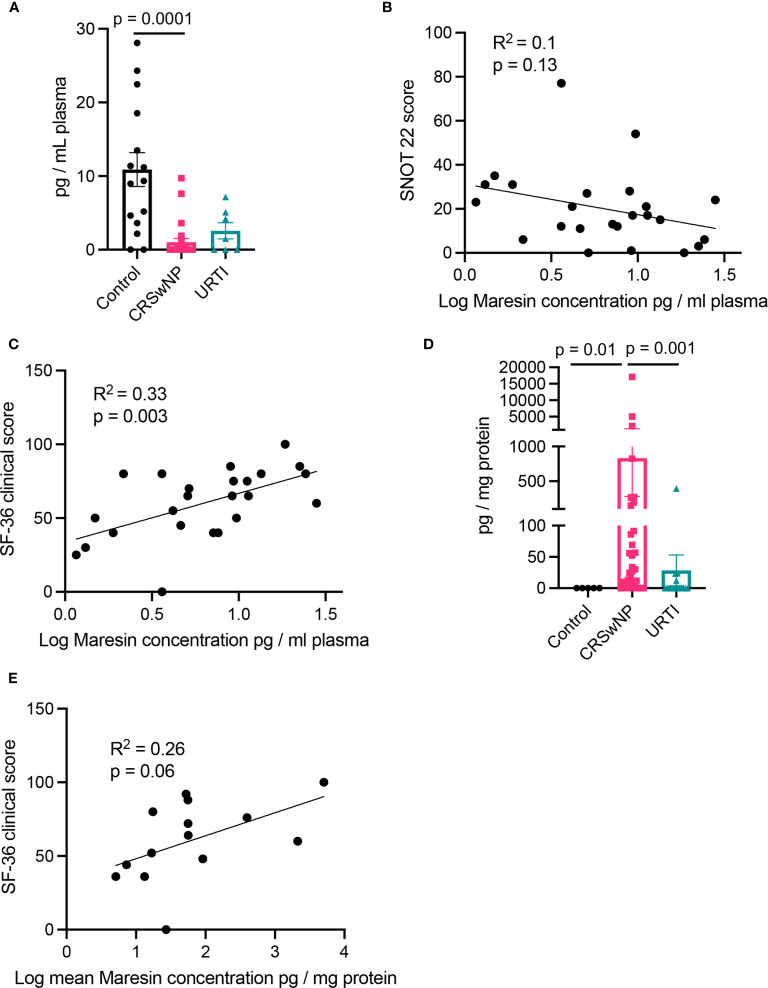
Plasma Maresin levels correlate with clinical scores. **(A)** Plasma Maresin levels (i.e., sum of MaR1, 7S,14S-diHDHA, MaR2, 22-OH-MaR1, 22-COOH-MaR1, 14-oxo-MaR1, 4,14-diHDHA, MaR1_n-3 DPA_, MaR2_n-3 DPA_ and 7S,14S-diHDPA) for healthy volunteers (n = 15), CRSwNP (n = 24), URTI (n = 8) patients. **(B, C)** Linear regression analysis of Maresins levels in plasma in relation to **(B)** SNOT 22 scores **(C)** 36-Item Short Form Health Survey general health measures. **(D)** Maresin concentrations in nasal secretions. **(E)** Linear regression analysis of Maresins levels in plasma in relation to 36-Item Short Form Health Survey general health measures. **(A, D)** Kruskal-Wallis test used to identify differences between the mean ranks of at least one pair of the groups. Dunn’s pairwise tests was carried out to identify differences between group pairs.

In plasma from all three groups, we identified mediators from the DHA and n-3 DPA maresin families that included the DHA-derived MaR1 and the n-3 DPA derived MaR2_n-3 DPA_ (**Table 2**). Assessment of overall plasma concentrations for this SPM family (i.e., the sum of MaR1, 7S,14S-diHDHA, MaR2, 22-OH-MaR1, 22-COOH-MaR1, 14-oxo-MaR1, 4,14-diHDHA, MaR1_n-3 DPA_, MaR2_n-3 DPA_ and 7S,14S-diHDPA) demonstrated a significant decreased in plasma concentrations of these mediators in the CRSwNP cohort when compared to healthy controls (**Figure 2A**). Furthermore, concentrations of this SPM family were observed to be negatively correlated with both SNOT 22 scores as well as SF-36 general health measures in patients with CRSwNP (**Figures 2B, C**). In nasal secretions we also identified members of this SPM family, including the DHA-derived MaR1 and MaR2 (**Table 2**). Notably, we observed that in nasal secretions, Maresin concentrations were significantly upregulated when compared with levels observed in secretions from both healthy volunteers and patients with a URTI (**Figure 2D**). We also observed that concentrations of these mediators were positively correlated with SF36 general health measures in patients with CRSwNP (**Figure 2E**).

**Table 2:** Plasma Lipid mediator concentrations.

	Control (pg/mL)			CRSwNP (pg/mL)				URTI (pg/mL)			
**DHA metabolome**	mean	±	sem	mean		±	sem	mean		±	sem
RvD1	0.05	±	0.06	0.12		±	0.08	1.17		±	0.41
17R-RvD1		–		0.04		±	0.03	0.21		±	0.21
RvD2	133.94	±	86.45	30.44		±	18.38	465.20		±	194.48
RvD3	0.03	±	0.02			–				–	
17R-RvD3	1.49	±	1.20	0.01		±	0.01			–	
RvD4		–				–				–	
RvD5	0.19	±	0.11	0.03		±	0.03	1.48		±	1.22
RvD6		–				–		0.18		±	0.18
PD1	0.29	±	0.25	0.61		±	0.27	0.78		±	0.58
10S,17S-diHDAH	0.59	±	0.35			–		0.66		±	0.43
22-OH-PD1	2.05	±	2.12			–				–	
17R-PD1	0.32	±	0.29	0.32		±	0.26	0.24		±	0.18
MaR1	2.25	±	1.33	0.75		±	0.41	1.95		±	0.97
7S,14S-diHDHA	0.84	±	0.73	0.18		±	0.18			–	
MaR2		–		0.08		±	0.06			–	
22-OH-MaR1	0.65	±	0.46			–				–	
22-COOH-MaR1		–				–				–	
14-oxo-MaR1		–				–				–	
4,14-diHDHA		–				–				–	
**n-3 DPA metabolome**											
RvT1		–		0.36		±	0.26			–	
RvT2	0.04	±	0.04	0.03		±	0.02	0.06		±	0.06
RvT3	0.07	±	0.07	1.29		±	0.95	0.53		±	0.26
RvT4	0.66	±	0.35	0.05		±	0.03	2.37		±	1.89
RvD1 _n-3 DPA_	0.55	±	0.57	1.26		±	1.01	0.20		±	0.20
RvD2 _n-3 DPA_	0.21	±	0.15	0.04		±	0.04			–	
RvD5 _n-3 DPA_	0.55	±	0.35	0.39		±	0.28			–	
PD1 _n-3 DPA_	0.90	±	0.87			–				–	
PD2 _n-3 DPA_		–				–				–	
10S, 17S-diHDPA	0.47	±	0.49			–				–	
22-OH-PD1_n-3 DPA_		–				–				–	
MaR1 _n-3 DPA_	0.09	±	0.09			–		0.61		±	0.40
MaR2 _n-3 DPA_	7.05	±	2.38			–				–	
7S,14S-diHDPA		–				–				–	
**EPA metabolome**											
RvE1	4.39	±	4.37	0.04		±	0.04			–	
RvE2		–		0.19		±	0.07	0.10		±	0.10
RvE3		–				–		1.41		±	1.41
**AA metabolome**											
LXA_4_		–		0.04		±	0.02	1.20		±	0.92
LXB_4_	5.76	±	4.44			–		5.69		±	5.69
5S,15S-diHETE	0.13	±	0.09	13.40		±	9.04			–	
13,14-dehydro-15-oxo-LXA_4_											
15-oxo-LX_4_				0.02		±	0.01	0.39		±	0.39
15-epi-LXA_4_	0.22	±	0.17	0.06		±	0.03	1.49		±	1.49
15-epi-LXB_4_	2.28	±	1.76	0.08		±	0.08	5.69		±	5.69
LTB_4_	7.96	±	4.97	7.56		±	1.87	8.53		±	3.84
5S,12S-diHETE	7.41	±	5.19	3.19		±	1.91	2.48		±	1.76
6-trans-LTB_4_	1.57	±	1.18	2.13		±	1.10	2.36		±	1.61
6-trans-12-epi LTB_4_	0.06	±	0.07	0.14		±	0.07			–	
20-OH-LTB_4_	0.00	±	0.00	0.94		±	0.42	0.59		±	0.59
20-COOH-LTB_4_	0.43	±	0.30	0.62		±	0.26	2.08		±	2.08
PGE_2_	2.47	±	1.60	4.24		±	0.96	5.43		±	2.34
PGD_2_	5.72	±	3.53	3.39		±	0.80	3.39		±	1.15
PGF_2a_	1.05	±	0.84	3.45		±	1.03	3.89		±	1.56
TxB_2_	28.68	±	23.94	71.19		±	28.47	29.88		±	15.95

Plasma was collected from patients with CRSwNP (n = 24) healthy volunteers (n =15) and subjects with URTI and LM were extracted using C18 SPE and LM concentrations determined using LC-MS/MS based LM profiling. - = Below limits.

### Distinct Lipid Mediator Profiles in Patients With CRSwNP When Compared to Healthy Volunteers and Patients With URTI

**Figure 3:**
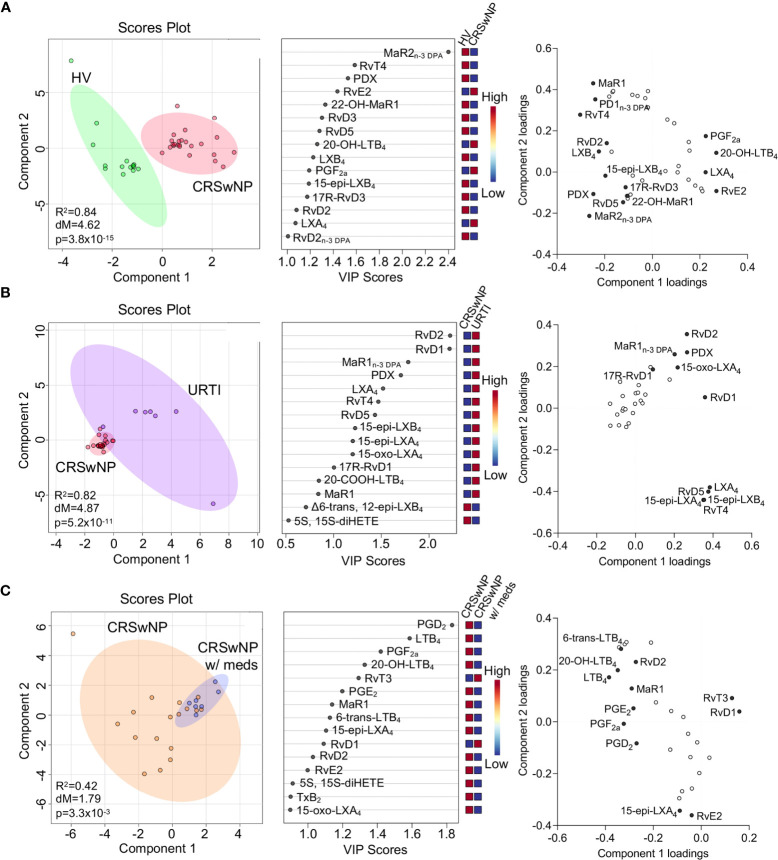
Dysregulated lipid mediator profiles in plasma from CRSwNP when compared to both healthy volunteers and URTI. **(A)** Plasma was collected from patients with CRSwNP (n = 24) and healthy volunteers (HV, n = 15) LM concentrations were determined using LC-MS/MS based LM profiling (see methods for details). Differences in LM concentrations were evaluated using PLS-DA. (*Left panel*) 2-dimensional scores plot and (*centre panel*) corresponding VIP plot of plasma LM. (*right panel*) loading plot where mediators with VIP score >1 are annotated. **(B)**. Plasma was collected from patients with CRSwNP (n = 24) and patients with URTI (n = 7) LM concentrations were determined using LC-MS/MS based LM profiling (see methods for details). Differences in LM concentrations were evaluated using PLS-DA. (*Left panel*) 2-dimensional scores plot and (*centre panel*) corresponding VIP plot of plasma LM. (*right panel*) loading plot where mediators with VIP score >1 are annotated. **(C)** Plasma lipid mediator concentrations were evaluated in patients receiving medication and those that were not. (*Left panel*) 2-dimensional scores plot and (*centre panel*) corresponding VIP plot of plasma LM. (*right panel*) loading plot where mediators with VIP score >1 are annotated. N = 7 for patients receiving medications (CRSwNP w/meds) and n = 17 for patients not receiving medications (CRSwNP). Shaded areas represent 95% confidence interval (CI), R^2^ = coefficient-of-determination, p = p-value from Hotelling’s T-squared test, dM = Mahalanobis distance between groups.

Having found changes in plasma Maresin concentrations in patients with CRSwNP we next set out to evaluate whether plasma concentrations of other SPM families together with those of the arachidonic acid (AA)-derived eicosanoids were differentially regulated in patients with CRSwNP when compared with those found in healthy volunteers. Using a targeted LC-MS/MS approach we identified mediators from all four essential fatty acid metabolomes, i.e., DHA, n-3 DPA, eicosapentaenoic acid (EPA) and AA, in plasma from patients with CRSwNP as well as in plasma from healthy volunteers (**Table 2**). To evaluate the relationship between peripheral blood LM concentrations we employed partial least squares discriminant analysis (PLS-DA). PLS-DA is a dimensionality-reducing multivariate analysis that creates a linear regression model accounting for multicollinearity and identifies the relationship between samples based on LM concentrations. Using PLS-DA we found that LM concentrations in plasma from participants with CRSwNP were distinguishable from those identified in healthy controls (**Figure 3A** and **Table 2**). The robustness of this model was evaluated by assessing the coefficient-of-determination (R^2^) which gave a value of 0.84. The separation between these two groups was statistically evaluated using the Mahalanobis distance between groups, which gave a value of 4.62, and Hotelling’s T-squared test which gave a p value of 3.8 x10^-15^ (**Figure 3A**). To assess which of the identified mediators contributed to the observed separation between the groups we assessed the variable importance in projection (VIP) scores that identified mediators contributing the most to the observed separation. Here we found that 9 mediators displayed a VIP score greater than 1. These included both pro-resolving, e.g., MaR2_n-3 DPA_ and RvT4, and pro-inflammatory, e.g. PGF_2a,_ autacoids. This analysis also highlighted an overall downregulation in SPM biosynthesis in plasma from patients with CRSwNP whereby out of the 13 SPM found to be differentially regulated between the two groups, 11 were found to be reduced in the CRSwNP. This was linked with an increase in both the pro-inflammatory and smooth muscle contracting agent PGF_2a_ and an increase in 20-OH-LTB_4_, the further metabolite of the potent leukocyte chemoattractant LTB_4._ These observations highlight a shift in plasma lipid mediator profile of patients with CRSwNP towards a pro-inflammatory profile.

We next assessed whether plasma LM concentrations were differentially regulated in patients with a URTI when compared to CRSwNP. Assessment of the clustering obtained with the lipid mediator profile from these patients demonstrated that plasma lipid mediator concentrations between the two patient groups was different, as observed from the distinct clustering (**Figure 3B**). This separation was observed to be statistically significant as demonstrated by a p value of 5.2x10^-11^ (**Figure 3B**). Evaluation of the VIP scores demonstrated that the differences observed were primarily related to 10 SPM, which included RvD2, RvD1, RvT4 and 15-epi-LXA_4_. Intriguingly the concentrations of all these mediators were upregulated in patients with URTI, in line with the clinical observation that this a self-limited inflammatory response (**Figure 3B**).

It is now appreciated that distinct drugs can impact directly or indirectly on SPM production. Therefore, we next evaluated whether those patients that at time of sample collection we receiving treatment displayed a distinct lipid mediator profile to those that were not receiving medication. Results from this analysis demonstrated that there was a statistically significant shift in the lipid mediator profiles between these two groups (**Figure 3C**). This shift in lipid mediator profiles was linked with the upregulation of lipid mediator production in patients that were not receiving medication at the time of sample collection. This suggests that the therapeutics being administered, which included topical and systemic steroids as well as antibiotics, may impact lipid mediator pathway in these patients.

### Nasal Secretions From Patients With CRSwNP Display an Altered Lipid Mediator Profile

**Figure 4:**
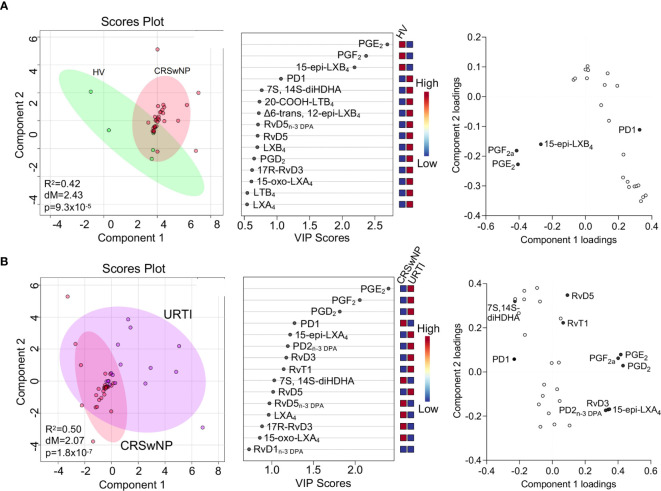
Nasal secretions from patients with CRSwNP display a distinct LM profile to those from both healthy volunteers and URTI patients. **(A)** Nasal secretions were collected from patients with CRSwNP (n = 16) and healthy volunteers (HV, n = 5) LM concentrations were determined using LC-MS/MS based LM profiling (see methods for details). Differences in LM concentrations were evaluated using PLS-DA. (*Left panel*) 2-dimensional scores plot and (*centre panel*) corresponding VIP plot of plasma LM (*right panel*) loading plot where mediators with VIP score >1 are annotated. **(B)** Nasal secretions were collected from patients with CRSwNP (n = 16) and patients with URTI (n = 8) LM concentrations were determined using LC-MS/MS based LM profiling (see methods for details). Differences in LM concentrations were evaluated using PLS-DA. (*Left panel*) 2-dimensional scores plot and (*centre panel*) corresponding VIP plot of plasma LM (*right panel*) loading plot where mediators with VIP score >1 are annotated. Shaded areas represent 95% confidence interval (CI), R^2^ = coefficient-of-determination, p = p-value from Hotelling’s T-squared test, dM = Mahalanobis distance between groups.

Having found that SPM concentrations were altered in plasma from patients with CRSwNP we next sought to evaluate whether this was also true for nasal secretions obtained from these patients in comparison with levels found in healthy volunteers. In these secretions we also identified mediators from the lipoxygenase and cyclooxygenase metabolomes of all four fatty acids, including the DHA-derived 22-OH-MaR1, n-3 DPA-derived MaR2_n-3 DPA_, EPA-derived RvE2 and AA-derived LXB_4_ (**Table 3**). PLS-DA analysis demonstrated a separation in the LM profiles of nasal secretions from patients with CRSwNP from those obtained from healthy volunteers (**Figure 4A**). This was primarily linked with an upregulation of PGE_2_ and PGF_2a_ in healthy volunteers compared to patients with CRSwNP (**Figure 4A**).

**Table 3:** Lipid mediator profiles from nasal secretions.

	Control (pg/mg protein)			CRSwNP (pg/mg protein)			URTI (pg/mg protein)		
**DHA metabolome**	mean	±	sem	mean	±	sem	mean	±	sem
RvD1		–			–		0.03	±	0.03
17R-RvD1		–			–			–	
RvD2		–			–			–	
RvD3		–			–		1.86	±	1.56
17R-RvD3		–		0.42	±	0.23		–	
RvD4		–			–			–	
RvD5	103.06	±	85.45	398.47	±	143.36	975.15	±	493.43
RvD6		–			–			–	
PD1		–		10.71	±	3.41	1.62	±	0.93
10S,17S-diHDAH	9.41	±	8.59	25.30	±	25.70	56.49	±	40.33
22-OH-PD1		–			–			–	
17R-PD1		–		0.91	±	0.70		–	
MaR1		–		788.95	±	559.54	26.22	±	25.47
7S,14S-diHDHA		–		26.74	±	11.86	1.57	±	1.19
MaR2		–		16.13	±	12.97	0.62	±	0.46
22-OH-MaR1		–			–			–	
22-COOH-MaR1		–			–			–	
14-oxo-MaR1		–			–			–	
4,14-diHDHA		–		0.33	±	0.34		–	
**n-3 DPA metabolome**									
RvT1		–			–		0.37	±	0.32
RvT2		–			–			–	
RvT3		–			–			–	
RvT4		–			–			–	
RvD1_n-3 DPA_		–		0.02	±	0.01		–	
RvD2_n-3 DPA_		–		0.04	±	0.04		–	
RvD5_n-3 DPA_		–		21.26	±	10.23	0.93	±	0.65
PD1 _n-3 DPA_	4.98	±	4.54		–			–	
PD2 _n-3 DPA_	44.61	±	40.72		–		29.21	±	23.99
10S, 17S-diHDPA		–		0.06	±	0.06	0.10	±	0.11
22-OH-PD1n _n-3 DPA_		–			–			–	
MaR1_n-3 DPA_		–			–			–	
MaR2_n-3 DPA_		–			–			–	
7S,14S-diHDPA		–			–			–	
**AA metabolome**									
RvE1		–			–			–	
RvE2		–			–			–	
RvE3	104.09	±	95.02	78.40	±	79.65	48.23	±	29.01
**AA metabolome**									
LXA_4_		–		3.22	±	2.00	149.68	±	154.44
LXB_4_		–		32.75	±	15.98	37.82	±	13.93
5S,15S-diHETE	146.84	±	134.05	96.91	±	98.46	135.93	±	84.12
13,14-dehydro-15-oxo-LXA_4_		–			–			–	
15-oxo-LXA_4_		–		1.57	±	0.88	0.04	±	0.03
15-epi-LXA_4_		–					2.46	±	1.97
15-epi-LXB_4_	263.41	±	240.46	0.15	±	0.16	0.00	±	0.00
LTB_4_	71.93	±	42.07	161.59	±	56.43	157.18	±	80.03
5S,12S-diHETE		–		0.32	±	0.32		–	
6-trans-LTB_4_	38.19	±	33.42	85.22	±	39.31	124.85	±	60.29
6-trans-12-epi-LTB_4_		–		94.39	±	44.69	52.73	±	49.82
20-OH-LTB_4_		–		501.80	±	373.60	26.16	±	21.46
20-COOH-LTB_4_		–		10.17	±	4.71	13.50	±	6.26
PGE_2_	1926.04	±	1110.20	265.65	±	54.78	1408.65	±	411.74
PGD_2_	1.81	±	1.65	37.97	±	19.05	489.73	±	236.24
PGF_2a_	718.47	±	501.80	96.36	±	18.92	434.53	±	148.98
TxB_2_	7.69	±	4.45	5.96	±	4.75	3.18	±	1.46
LTC_4_		–			–			–	
LTD_4_		–			–			–	
LTE_4_		–		9.73	±	2.95	2.55	±	1.45

Plasma was collected from patients with CRSwNP (n = 16) healthy volunteers (n = 5) and subjects with URTI (8) and LM were extracted using C18 SPE and LM concentrations determined using LC-MS/MS based LM profiling. - = Below limits.

To gain further insights into the regulation of LM at the site of inflammation, we next compared nasal secretion LM profiles from patients with URTI. Here we observed a separation in the cluster representing URTI patients when compared with that representing LM profiles from patients with CRSwNP (**Figure 4B**). The separation in these clusters was primarily linked with the differential regulation of 12 lipid mediators that included the pro-resolving mediators RvD5 and RvD5_n-3 DPA_ and pro-inflammatory eicosanoids, including PGE_2_ and PGF_2a_. Assessment of relative levels of these mediators demonstrated an upregulation of PGD_2_, PGE_2_ and PGF_2a_ in patients with URTI together with that of a number of pro-resolving mediators including 15-epi-LXA_4_, PD2_n-3 DPA_ and RvD3 (**Figure 4B**).

### Maresin 1 Regulates CRSwNP Circulating Monocyte Activation

**Figure 5:**
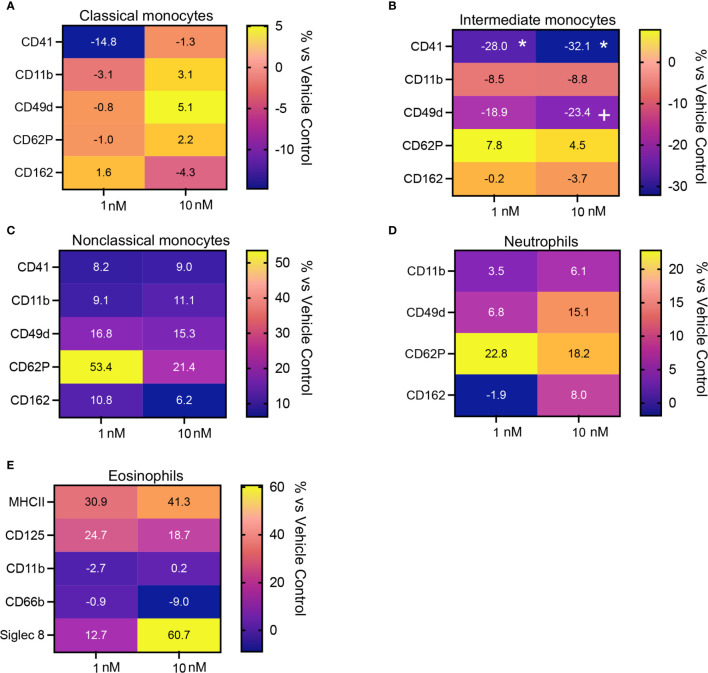
MaR1 regulates the expression of activation markers on peripheral blood phagocytes from patients with CRSwNP. **(A–E)** peripheral blood from CRSwNP patients was incubated with vehicle or MaR1 (1 or 10nM) for 45 minutes at 37°C and the expression of the indicated activation markers was evaluated on **(A)** CD14++CD16+ Classical monocytes, **(B)** CD14++CD16++ Intermediate monocytes, **(C)** CD14+CD16++ Non-Classical monocytes, **(D)** CD16+ neutrophils and **(E)** Siglec 8+ eosinophils using flow cytometry. Results are means of percentage (%) change from values obtained in vehicle controls for each patient. N = 7 patients for **(A–D)** and 10 patients for **(E)**. *p < 0.05.

Having observed that Maresin concentrations were significantly reduced in plasma from patients with CRSwNP and given that circulating phagocytes become activated in chronic inflammatory conditions we next assessed whether addback of MaR1, which exerts potent leukocyte-directed activities (31), may regulates phagocyte activation in peripheral blood cells from these patients. Incubation of peripheral blood cells with MaR1 led to a down regulation in the expression of activation markers on classical and intermediate monocytes and eosinophils reaching statistical significance in intermediate monocytes for CD41 expression, a marker of platelet-monocyte activation (**Figures 5A–E** and **Supplementary Figures 1**, **2**).

**Figure 6:**
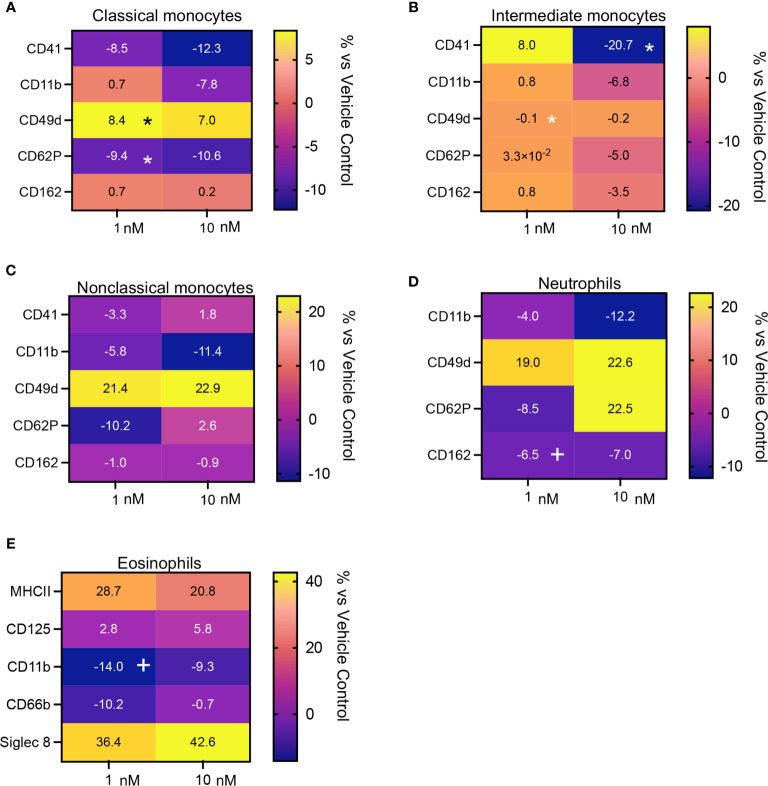
Regulation of activation marker expression in peripheral blood from CRSwNP patients incubated with PAF by MaR1. Peripheral blood from CRSwNP patients was incubated with vehicle or MaR1 (1 or 10nM) for 15 minutes at 37°C then with PAF 100 (nM) for a further 30 minutes at 37°C and the expression of the indicated activation markers was evaluated on **(A)** CD14++CD16+ Classical monocytes, **(B)** CD14++CD16++ Intermediate monocytes, **(C)** CD14+CD16++ Non-Classical monocytes, **(D)** CD16+ neutrophils and **(E)** Siglec 8+ eosinophils using flow cytometry. Results are means of percentage (%) change from values obtained in vehicle controls for each patient. N = 7 patients for **(A–D)** and 10 patients for **(E)**. *p < 0.05.

We next evaluated whether the leukocyte directed activities of MaR1 were retailed in the presence an inflammatory challenge. For this purpose, we incubated whole blood with platelet activating factor (PAF) together with MaR1. Here we found that MaR1 decreased the expression of activation markers, including CD41, CD11, CD62P and CD162 on monocyte subsets, neutrophils, and eosinophils (**Figures 6A–E** and **Supplementary Figures 1**, **3**) reaching statistical significance for CD62P on classical monocytes and CD41 on intermediate monocytes. We also observed a significant upregulation of CD49d expression on classical monocytes following MaR1 incubation (**Figures 6A–D**). Together these findings indicate that MaR1 regulates the expression of phagocytes from patients with CRSwNP.

## Discussion

In the present study we explored whether disruption in SPM production, and in particularly that of Maresins, were linked to disease in patients with CRSwNP. In peripheral blood from patients with CRSwNP we observed a significant downregulation in concentration of Maresins when compared with healthy volunteers. These changes were linked with an overall downregulation of other SPM in plasma from these patients when compared with concentrations observed in healthy volunteers and patients with URTI. Interestingly, we found that Maresin concentrations in nasal secretions from these patients were significantly upregulated when compared to both healthy volunteers and patients with URTI. Of note, nasal secretion, and plasma Maresin concentrations were positively correlated with SF36 scores in patients with CRSwNP. Furthermore, incubation of peripheral blood with MaR1 reduced circulating phagocyte activation as evidenced by a downregulation of adhesion molecule expression.

It is estimated that 100 million people worldwide are affected by chronic rhinosinustits^4^. Longitudinal data from the Clinical Practice Research Datalink suggests that 1% of British adults receive treatment each year in primary care, resulting in multiple GP consultations and medical prescriptions (32). In the secondary care setting, this translates into 120,000 out-patient consultations and 40,000 operations in England and Wales annually (7). The difficulties in initiating and maintaining appropriate treatments for CRSwNP are reliant upon an accurate diagnosis, which in the primary care setting is challenging as the symptoms associated with CRSwNP (nasal obstruction, discharge, loss of smell and facial pain) are identifiable in all nasal cavity ailments. In addition, limited ENT training for medical professionals (33) and lack of diagnostic equipment available in primary care are recognised contributing factors (32). In the present studies we observed that LM profiles in both plasma and nasal secretions from patients with CRSwNP were distinct from those obtained in both healthy volunteers and patients with URTI. Our findings are also in accord with recent studies demonstrating that lipid mediator concentrations were different in sinus tissues from patients with CRSwNP when compared to both control subjects and CRS patients without polyps (21).

Adhesion molecules play a central role in phagocyte recruitment into tissues as well as in the formation of platelet-phagocyte heterotypic aggregates which are involved in the propagation of both systemic and airway inflammation (31, 34). Flow cytometric assessment of peripheral blood phagocytes demonstrated that MaR1 displayed potent phagocyte-directed actions regulating the expression of adhesion molecules, including CD162 and CD11b on neutrophils and eosinophils, two proteins involved in the recruitment of phagocytes into inflamed tissues. Furthermore, we observed that MaR1 significantly downregulated the expression of markers for platelet-leukocyte heterotypic aggregates, namely CD41 and CD62P, on classical and intermediate monocytes, with a trend for the reduction in the expression of CD41 also observed in neutrophils and non-classical monocytes. These heterotypic aggregates are linked with both an increased phagocyte recruitment and activation in various acute and chronic inflammatory conditions, including COVID-19 (35). Together these observations are also in accord with previous studies demonstrating that MaR1 regulates leukocyte trafficking to the site of inflammation.

The integrated approach employed in the present studies evaluating lipid mediator profiles in both peripheral blood and tissues and linking these to quality-of-life scores and phagocyte responses provides novel insights into the role of SPM in the pathophysiology of CRSwNP. There are a number of limitations that should be noted, the first being that due to the small number of patients recruited we were unable to evaluate the impact of different drugs on lipid mediator production in patients with CRSwNP. This is of relevance given that in our analysis we observed that patients receiving some form of medication displayed distinct lipid mediator profiles when compared with those not receiving medication at time of study enrolment. Therefore, future studies will need to evaluate the impact that distinct treatments have on lipid mediator production and how this links with outcome. A second limitation of this study is that patients with CRSwNP enrolled in this study were on average older than those with URTI. Since SPM production is reduced with age this might have potentially contributed to the higher SPM levels reported in patients with URTI. Thus, future studies comparing these two groups would need to exclude the impact of age on the observed differences in SPM production observed herein. Another aspect that requires further evaluation is the mechanisms leading to the alteration of maresin production in both peripheral blood and tissues and whether maresin signalling and function in extravasated leukocyte is impaired, contributing to dysregulated phagocyte responses.

In summation, in the present studies we observed that lipid mediator concentrations in both plasma and nasal secretions from patients with CRSwNP were markedly different to those obtained from either healthy volunteer and patients with a URTI. We also found that cumulative Maresin concentrations were markedly reduced in plasma from patients with CRSwNP whilst being significantly increased in nasal secretions when compared to both healthy volunteers and patients with URTI. Furthermore, concentrations of these mediators were correlated with quality-of-life scores supporting the hypothesis that lipid mediators, potentially Maresins, may be useful diagnostics in patients with CRSwNP. We also observed that MaR1 regulated phagocyte activation suggesting that pro-resolving therapeutics may be useful in limiting chronic inflammation in these patients.

## Data Availability Statement

The original contributions presented in the study are included in the article/**Supplementary Material**. Further inquiries can be directed to the corresponding author.

## Ethics Statement

The studies involving human participants were reviewed and approved by NHS Research Ethics Committee. The patients/participants provided their written informed consent to participate in this study. Participants with a history of CRSwNP with failed medical management, healthy controls with no previous ear, nose and throat disease as well as those undergoing nasal cavity surgery due to non-inflammatory conditions (cosmetic or post traumatic deformity; septoplasty or septorhinoplasty) and participants with URTI were recruited. (Research Ethics Committee Number: 17/EM/0447).

## Author Contributions

JD and IB designed the experiments. IB and DSK conducted the experiments and/or analysed results. All authors contributed to the article and approved the submitted version. JD and GA contributed to supervision of the work JD, GA, and IB conceived overall research plan.

## Funding

JD received funding from the European Research Council (ERC) under the European Union’s Horizon 2020 research and innovation programme (grant no: 677542) and the Barts Charity (grant no: MGU0343). JD is also supported by a Sir Henry Dale Fellowship jointly funded by the Wellcome Trust and the Royal Society (grant 107613/Z/15/Z).

## Conflict of Interest

JD is Scientific founder and director of Resolomics Ltd and Inventor on several patents and patent applications assigned to Brigham and Women’s Hospital (Boston, MA) and Queen Mary University of London (London, UK) for the therapeutic and diagnostic use of specialized pro-resolving mediators.

The remaining authors declare that the research was conducted in the absence of any commercial or financial relationships that could be construed as a potential conflict of interest.

## Publisher’s Note

All claims expressed in this article are solely those of the authors and do not necessarily represent those of their affiliated organizations, or those of the publisher, the editors and the reviewers. Any product that may be evaluated in this article, or claim that may be made by its manufacturer, is not guaranteed or endorsed by the publisher.
