# Supplementary figures and images for: Dysregulated Maresin Concentrations in Plasma and Nasal Secretions From Patients With Chronic Rhinosinusitis
Source: Front Immunol. 2021 Aug 31;12:733019. doi: 10.3389/fimmu.2021.733019 (PMC8438229; doi:10.3389/fimmu.2021.733019)

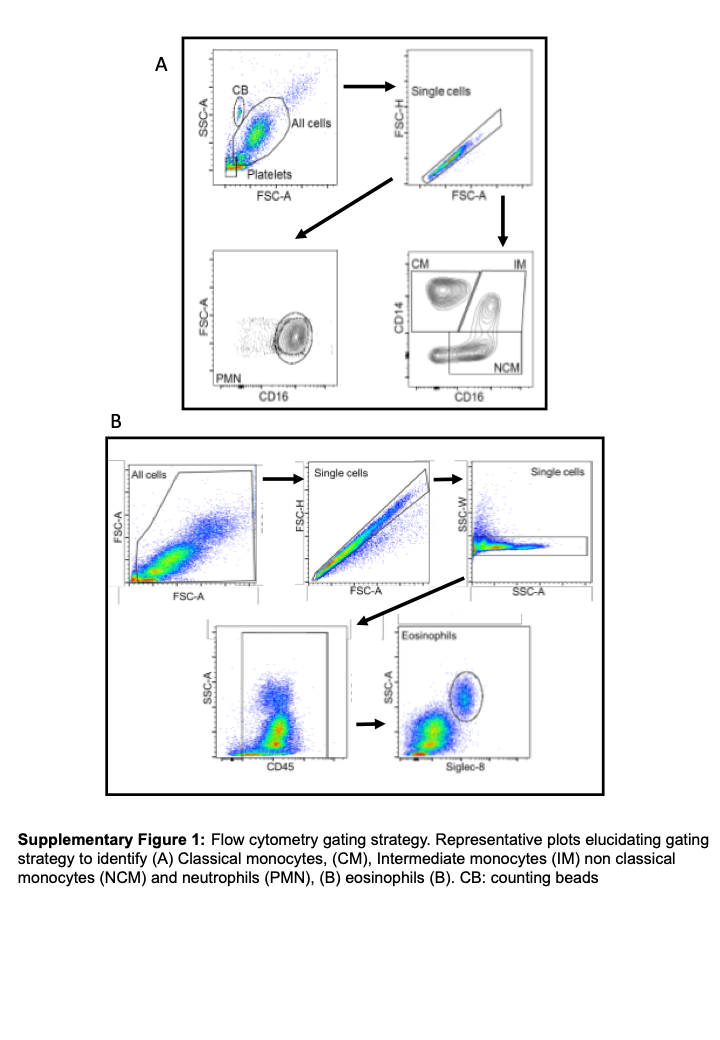

Supplement: Supplementary file 1 [file Image_1.tiff]

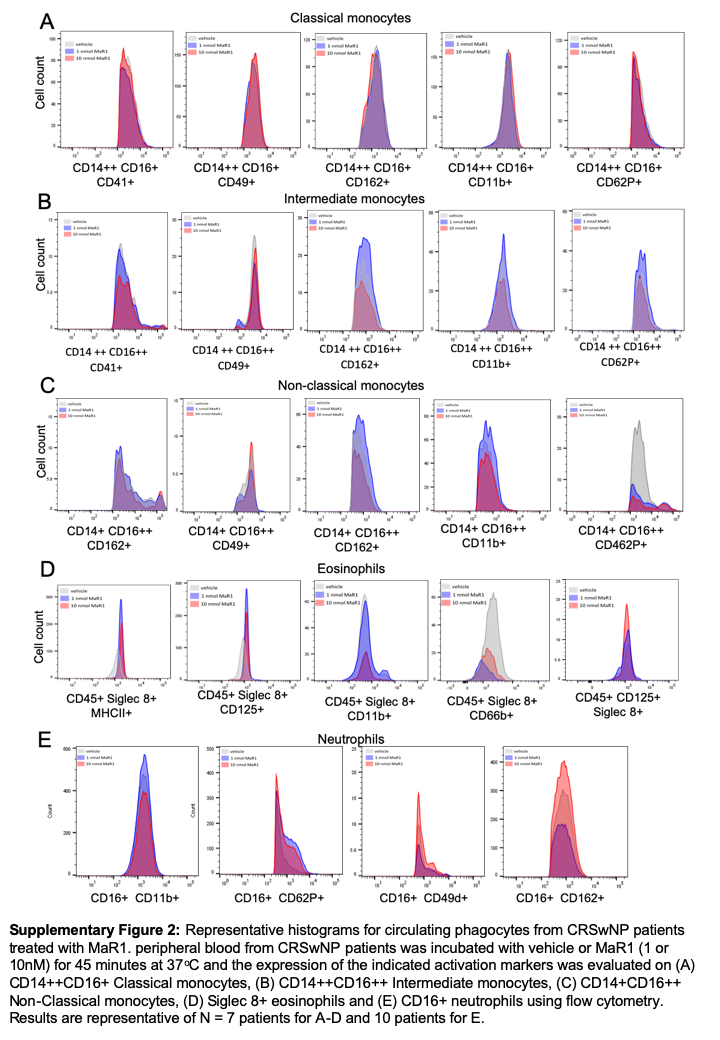

Supplement: Supplementary file 2 [file Image_2.tiff]

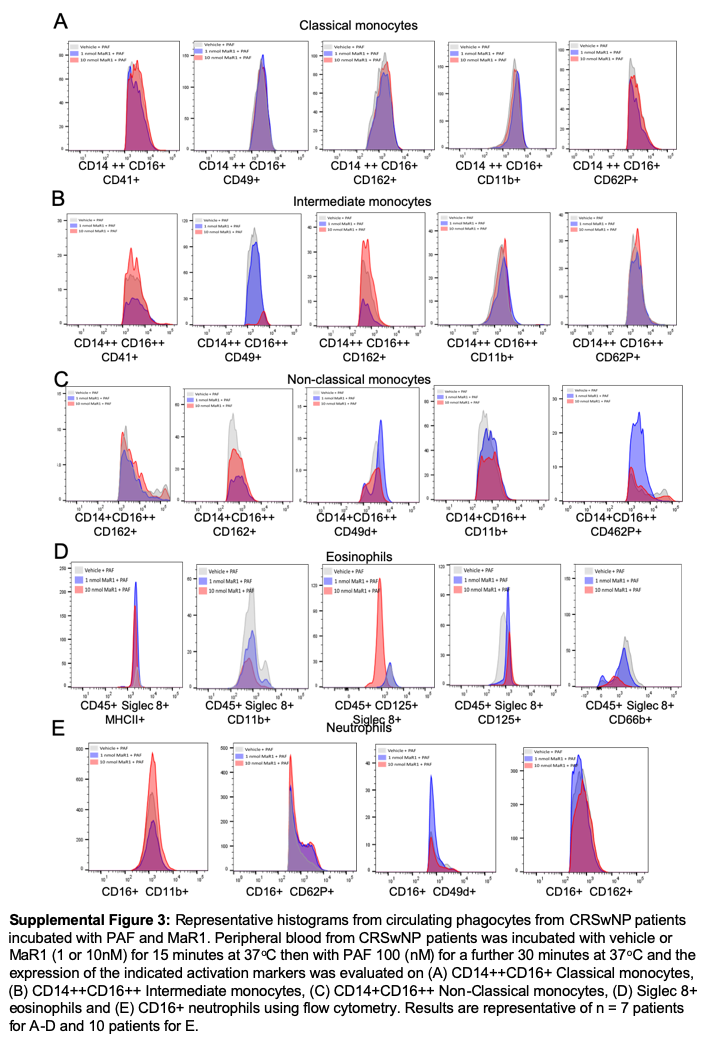

Supplement: Supplementary file 3 [file Image_3.tiff]
